# Can Healthy Checkout Counters Improve Food Purchases? Two Real-Life Experiments in Dutch Supermarkets

**DOI:** 10.3390/ijerph17228611

**Published:** 2020-11-19

**Authors:** Marlijn Huitink, Maartje P. Poelman, Jacob C. Seidell, Lothar D. J. Kuijper, Trynke Hoekstsra, Coosje Dijkstra

**Affiliations:** 1 Department of Health Science, Faculty of Science, Vrije Universiteit Amsterdam, Amsterdam Public Health Research Institute, De Boelelaan 1085, 1081 HV Amsterdam, The Netherlands; j.c.seidell@vu.nl (J.C.S.); l.d.j.kuijper@vu.nl (L.D.J.K.); trynke.hoekstra@vu.nl (T.H.); coosje.dijkstra@vu.nl (C.D.); 2 Chair Group Consumption and Healthy Lifestyles, Wageningen University & Research, Hollandseweg 1, 6706 KN Wageningen, The Netherlands; maartje.poelman@wur.nl

**Keywords:** supermarkets, checkout counter, purchase behavior, food purchases, snacks, impulsive behavior, food environment

## Abstract

Most snacks displayed at supermarket checkouts do not contribute to a healthy diet. We investigated the effects of introducing healthier snack alternatives at checkouts in supermarkets on purchasing behavior. In Study 1, we investigated the effect of completely substituting less healthy with healthier snacks (one supermarket). In Study 2, we investigated the effect of placing and discounting healthier snacks while the less healthy snacks remain in place (two supermarkets). In both studies, the number of purchased snacks (per 1000 customers) was used as the outcome variable. Results for Study 1 showed that the absolute number of purchased checkout snacks was 2.4 times lower (95% confidence interval (CI): 1.9–2.7) when healthier snacks instead of less healthy snacks were placed at the supermarket checkouts. Results for Study 2 showed that when additional healthier snacks were placed near the checkouts, the absolute number of healthier purchased snacks increased by a factor of 2.1 (95% CI: 1.3–3.3). When additional healthier snacks were placed near the checkouts and discounted, the absolute number of healthier purchased snacks increased by a factor of 2.7 (95% CI: 2.0–3.6), although this was not statistically significant higher than placement only (ratio: 1.1, 95% CI: 0.7–1.9). Purchases of less healthy snacks did not decline, and even slightly increased, during the intervention period (ratio: 1.3, 95% CI: 1.1–1.5). If supermarkets want to promote healthier snack purchases, additional healthier products can be positioned near the checkouts. However, this does not discourages the purchase of less healthy snacks. Therefore, to discourage unhealthy snack purchases at supermarket checkouts, a total substitution of less healthy snacks with healthier alternatives is most effective.

## 1. Introduction

Supermarkets are an important setting for food purchases, as they are the primary source of food and drinks for many people in high-income countries, and are becoming increasingly important in low- and middle-income countries [1,2,3,4]. In the Netherlands, around 80% of food purchases are made in supermarkets [5]. For this reason, supermarkets have a major influence on food choices, and in turn human diets [6,7,8,9,10,11]. Food purchases in supermarkets are influenced by several contextual factors within the supermarket, as many purchase decisions are made in-store [12]. According to the dual process theory, food decisions are steered by so called System 1, which is automatic and fast and System 2, which is deliberate and slow [13]. Supermarket customers are mostly unaware of in-store influences in supermarkets as they target unconscious decisions (e.g., via System 1) [14,15]. It has been estimated that more than half of in-store purchases in supermarkets are made impulsively, which are steered by various marketing techniques focusing on the placement, price and promotion of products [16,17,18].

As indicated by multiple studies [19,20,21,22,23], price discounts on healthy foods are an effective strategy for promoting healthier food purchases in supermarkets since price is one of the most important factors influencing food purchases, especially amongst people from groups with a lower socioeconomic position (SEP) [24]. Previous studies have also shown that placing and promoting healthier foods at key in-store locations (e.g., at ends-of-aisles, island bins) may trigger the purchase of these products [25,26]. Techniques to increase the likelihood that customers will make impulse purchases are predominantly used for the promotion of less healthy foods and drinks [3,27,28,29] despite evidence that these strategies can also be used to promote healthier food choices in supermarkets [26,30,31]. Therefore, using these strategies supporting healthier impulsive food choices has been encouraged by (inter)national health agencies since the consumption of energy-dense, ultra-processed foods and drinks has increased in many countries in recent decades [32,33]. The increase in consumption of these foods and drinks has contributed to an increase in overweight, obesity and other diet-related chronic diseases [34,35].

Checkout counters in supermarkets are an unavoidable point in supermarkets, and they are characterized by high levels of impulsive food purchases [36,37]. Many of the products that are sold at these counters are typical impulse products that not contribute to a healthy diet (e.g., candy bars). Moreover most supermarket checkouts lack the availability of products that are in favor of a healthy diet (e.g., fruit and vegetables) [38]. Various intervention strategies to optimize the healthiness of the food assortment at the checkouts in supermarkets have been evaluated. However, prior studies predominantly investigated the effects of the substitution of some less healthy snacks with healthier alternatives at checkouts [39,40,41]. Moreover, our previous study showed that the placement of additional healthier snacks at supermarket checkouts did not lead to the substitution of less healthy snack purchases with healthier alternatives [42]. The effect of completely substituting less healthy snacks with healthier alternatives at all of the checkout counters in supermarket has yet to be investigated. To date, it is unknown whether additional price discounts on healthier snacks placed near checkout counters could steer customers towards making healthier food purchases at supermarket checkout counters.

Therefore, the research reported in this article consisted of two studies investigating the effects of the introduction of healthier snacks at checkout counters on the number of purchased checkout snacks in Dutch supermarkets in an urban area with a lower SEP. In the first study, we examined the effect of completely substituting less healthy snacks with healthier alternatives at checkouts. In the second study, we investigated the effect of placing additional healthier snacks at checkouts (placement intervention), as well as the effect of offering a price discount on these healthier snacks (placement + price intervention) while allowing the less healthy snacks to remain for sale at checkouts during both interventions. We hypothesized that placing and discounting additional healthier snacks at the checkouts encourage customers to substitute less healthy with the healthier alternatives at the checkouts.

## 2. Materials and Methods

Both studies were conducted as part of a collaboration between the Amsterdam Healthy Weight Programme [43,44], the Amsterdam Health and Technology Institute (AHIT), the Albert Heijn—the supermarket chain with the largest market share in the Netherlands- and the Vrije Universiteit Amsterdam in the Netherlands. The overall aim of this collaboration was to create a healthier food environment for families in neighborhoods with a lower SEP in Amsterdam and to study the effectiveness of these efforts. In these neighborhoods, the prevalence of overweight and obesity is higher [45]. The Medical Ethics Committee of Vrije Universiteit Amsterdam confirmed that both studies were not subject to the Medical Research Involving Human Subjects Act (WMO), due to the nature of the measurements (anonymous sales data). The need for approval was therefore waived.

### 2.1. Supermarket Selection

For both studies, the headquarters of the supermarket chain selected and approached supermarkets in an area with a lower SEP in the southeastern part of Amsterdam, the Netherlands. This area was selected based on the Valuation of Immovable Property Act (VIPA), which is estimated annually [46]. This Act specifies how municipalities are to assess the value of homes and businesses within specific neighborhoods. A supermarket that was situated in a neighborhood with a very low VIPA was selected for this study. To recruit the supermarkets, store managers of the supermarkets were contacted by phone and informed about the study, while their participation was requested by an employee from the headquarters. After the store manager agreed to participate, the supermarket was visited to become acquainted with the store and the manager, while practical information about the study was provided by the employee from the headquarters and the researcher.

### 2.2. Categorization of Checkout Products

To assess the healthiness of all foods and drinks in both studies, we used the Wheel of Five criteria of the Netherlands Nutrition Centre, identifying products that contribute to a healthy diet for the Dutch population [47]. For the purpose of this study, checkout snacks were categorized as ‘healthier’ or ‘less healthy’, using the “Do I Choose Healthy?” app that integrates the criteria of the Wheel of Five [47]. If a specific product was not available in the mobile application, the nutrition facts label derived from the supermarket website was examined and manually classified by the researchers based on criteria of the Wheel of Five.

### 2.3. Study 1

In the first study, we examined the effect of completely substituting less healthy snacks with healthier snacks at checkout counters.

#### 2.3.1. Design and Setting

The first study was conducted in one supermarket (total number of checkouts: 8, annual number of customers: 1,768,000, size: 2300 m^2^) between September and December 2015. We used a quasi-experimental pre-post design with an eight-week control and an eight-week intervention period. During the control period, the usual less healthy snacks were offered at the checkout counters as usual. During the intervention period, less healthy snacks were completely substituted with healthier snacks. To examine the effect of this substitution we examined the sales data of snacks sold at the checkouts (which included the sales data of less healthy snacks during the control period and the sales data of the healthier snacks during the intervention period).

#### 2.3.2. Intervention

For the first study, the entire assortment of less healthy snacks displayed beside the conveyor belts was removed from all of the checkout counters and replaced with healthier single-pack snacks. In addition, displays of single-pack healthier snacks and ready-to-eat (e.g., fresh and pre-packed) fruit and vegetables were placed at the ends of the conveyor belts at 25% of the checkouts. The healthier snacks, which were selected by the supermarket chain, consisted of well-selling ready-to-use products (e.g., pieces of fruit, pre-packed vegetables, bottled water) that had already been sold before the Healthy Checkout Counter (HCC) intervention, as well as newly introduced healthier snack items (e.g., nut bars, cereal bars, smoothies, sliced and pre-packed fruit and vegetables), specifically developed for the Healthy Checkout Counter (HCC) intervention by the supermarket chain’s food suppliers. In all, 38 less healthy snacks were offered for sale exclusively at the checkouts during the control period, and they were replaced with 28 healthier snacks during the intervention period. The number of healthier snacks was based on the space available in the displays at the checkouts.

#### 2.3.3. Outcome Measures

Weekly sales data of the products that were offered at the checkout counters were provided by the participating supermarket. The main outcome measures were the weekly absolute number of snacks purchased per 1000 customers at the checkout counters (for less healthy snacks during the control period and for healthier snacks during the intervention period). The weekly number of purchased snacks per 1000 customers were calculated based on the total number of customers during that week. Given that we were interested only in the effect of the intervention on snacks that were sold exclusively at the checkout counters, products that were also displayed in other locations in the supermarket were excluded (7 products).

#### 2.3.4. Statistical Analyses

Descriptive statistics were used to examine the total number of purchased snacks at the checkout counters during the control period (less healthy snacks) and during the intervention period (healthier snacks). Because the sales data were not normally distributed, they are presented as medians with interquartile ranges (IQR). Sales volumes (i.e., the number of items sold) differ considerably across the snacks used in this study. It is thus not possible to make any meaningful comparison across these snacks. To examine the extent of the intervention’s effect on sales, we standardized the sales data for the snacks in order to normalize the distribution by taking the logarithms of their sales. The data were analyzed using the natural logarithms to calculate the proportional change in sales between the intervention and control period. We subsequently conducted an independent-sample t-test to investigate the proportional change in checkout sales between the intervention and control period. Log-transformed results were back-converted to ratios for presentation and interpretation. The outcome represents the ratio between the geometric means of the intervention period, as compared to the control period. Statistical analyses were performed using the statistical software package IBM SPSS Statistics for Windows, version 25.0.

#### 2.3.5. Results

**Figure 1:**
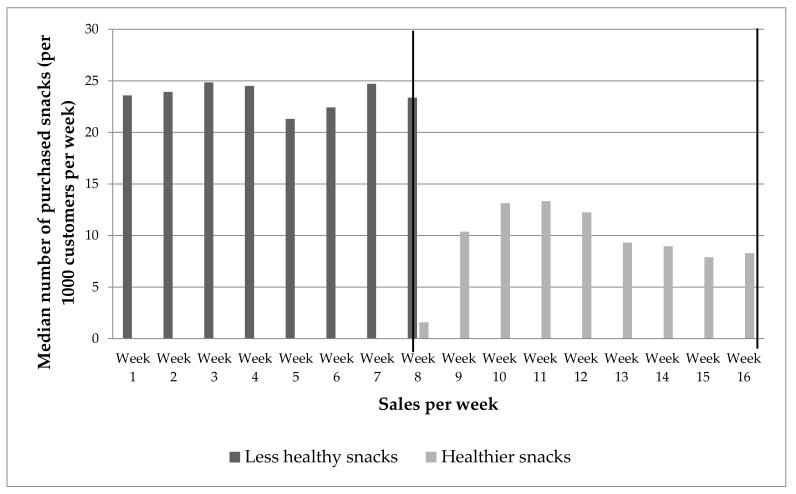
Median number of purchased snacks (per 1000 customers per week) of less healthy snacks and healthier snacks at checkout counters. The vertical lines mark the start and the end of the intervention period.

During the control period, the median weekly number of purchased less healthy snacks was 24 items (IQR: 2.8) per 1000 customers. During the intervention period, the median number of purchased healthier snacks was 10 (IQR: 4.5) items per 1000 customers (Figure 1). The absolute number of purchased checkout snacks (less healthy snacks in the control period vs. healthier snacks in the intervention period) was 2.4 (SE: 1.1, 95% CI: 1.9–2.7; t(8) = 11.0) times lower during the intervention period, as compared to the control period.

### 2.4. Study 2

In the second study, we investigated the effect of placing additional healthier snacks at checkouts as well as the effect of offering an additional price discount for the healthier checkout snacks, while keeping the usual less healthy checkout snacks available for sale.

#### 2.4.1. Design and Setting

The second study was conducted in two supermarkets that were different from the supermarket in Study 1 (Supermarket 1: total number of checkouts: 4, annual number of customers: 442,000, size: 1247 m^2^, Supermarket 2: total number of checkouts: 8, annual number of customers: 1,560,000, size: 1450 m^2^) between April and June 2017. We used a quasi-experimental pre-post design with a two-week control period, followed by a six-week intervention period. During the control period, less healthy snacks were offered at the checkout counters as usual. During the intervention period, the less healthy snacks remained for sale at the checkouts but additional healthier snacks were offered at the checkouts, either with or without a price discount of approximately 15%. To investigate the effect of the placement of additional healthier snacks at the checkouts and the price discount on the healthier snacks, we examined sales data of the healthier and less healthy snacks during the control and the intervention period.

#### 2.4.2. Intervention

The intervention of the second study consisted of placing three displays at the ends of the conveyor belts in front of the checkout counters, offering single packages of healthier snacks that had already been sold in the two supermarkets before the intervention. The less healthy snacks remained in their usual place at the checkout counters and were offered (not with a discount) during the entire intervention period. There were two types of interventions: (1) a placement intervention, in which additional healthier snacks were offered at the checkout counter and (2) the placement + price intervention, in which the healthier checkout snacks were offered at an additional price discount. The placement intervention and the placement + price intervention where alternated between the two supermarkets during the six-week intervention period (see Table 1). During the six-week intervention period, the assortment of healthier snacks was changed every two weeks, resulting in three consecutive two-week periods offering different types of healthier snacks including (1) vegetable snacks (tomatoes and cucumbers), (2) unsalted nuts (five different types) and (3) vegetable snacks (tomatoes and bell peppers) (Table 1). We conducted separate comparisons of the sales of the three types of healthier snacks during each two-week period to the sales during the two-week control period for the placement intervention and the placement + price interventions. The healthier snacks were selected by the research team and met the guidelines of the Dutch Nutrition Centre for a healthy diet [47].

#### 2.4.3. Outcome Measures

Daily sales data of the products that were offered at the checkout counters (less healthy and healthier snacks) were provided by the participating supermarket. The main outcome measures were the absolute number of both the less healthy snacks and the healthier snacks purchased per 1000 customers per day. The number of purchased less healthy snacks per 1000 customers was calculated based on the total number of customers during that day. The number of purchased less healthy snacks refers to the snacks that were offered exclusively at the checkout counters. The number of purchased healthier snacks refers to products that were placed at the checkout counters, as well as in another places in the supermarket (e.g., in the vegetable department). This was because the supermarket chain was not able to separate the sales data for products that were placed in two (or more) locations in the supermarket. In all, the study addressed 94 types of less healthy snacks and 9 types of healthier snacks.

#### 2.4.4. Compliance

During the intervention period, one of the researchers (M.H.) assessed the extent to which the intervention was being implemented as intended in the two supermarkets throughout the six-week period by making unannounced weekly visits to the intervention supermarkets and by contacting the managers of the supermarkets by telephone. During each visit, the researcher recorded compliance with the strategies (placement vs. placement + price) and documented the implementation of the intervention with photographs. This resulted in a list of days on which the intervention had been executed incorrectly (10 days).

#### 2.4.5. Statistical Analyses

Descriptive statistics were used to examine number of purchased less healthy and healthier snacks during the control and intervention periods. Because the sales data were not normally distributed, they are presented as medians with IQR. For the reasons described in the first study, we examined the extent of the effect of the intervention on the number of purchased snacks by standardizing the sales data for the snacks included in this study by using their logarithms. The data were analyzed using the natural logarithms to calculate the proportional changes in sales between the intervention and control periods. Because the sales data included zero values, we added a constant value to the data prior to applying the log transformation [48]. We subsequently conducted independent-sample t-tests to investigate the proportional change in sales between the intervention and control periods for the sales of (1) healthier snacks in the placement intervention, (2) healthier snacks in the placement + price intervention and (3) less healthy snacks. An additional independent-sample t-test was conducted to investigate the proportional change between the intervention and control periods in the number of purchased healthier snacks between the placement condition and the placement + price condition. Log-transformed results were back-converted to ratios for presentation and interpretation. The outcome represents the ratio between the geometric means of the intervention period as compared to the control period. The same statistical procedure was applied when analyzing the data according to the per-protocol approach, in order to investigate the effect of the intervention excluding sales data for the healthier snacks from ten days on which the intervention was not executed correctly [49]. Statistical analyses were performed using the statistical software package IBM SPSS Statistics for Windows, version 25.0.

#### 2.4.6. Results

**Figure 2:**
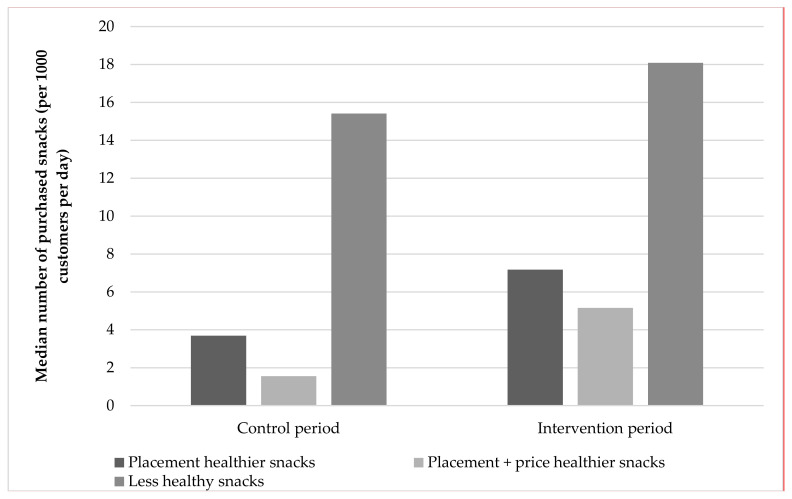
Median daily number of purchased snacks (per 1000 customers per day) of less healthy snacks, and healthier snacks separately during the placement intervention and healthier snacks during the placement + price intervention, during the in the control and intervention periods.

The median number of daily purchased healthier snacks per 1000 customers in the placement intervention increased from 4.2 (IQR: 4.6) items in the control period to 7.8 (IQR: 4.6) items in the intervention period (Figure 2). The absolute number of daily purchased healthier snacks per 1000 customers during the placement intervention was 2.1 times higher (SE: 1.3, 95% CI: 1.3–3.3; t(53) = 3.2, *p* < 0.001), as compared to the control period (Table 2). In line with these results, the median number of daily purchased healthier snacks per 1000 customers during the placement + price intervention increased from 2.2 (IQR: 4.7) items in the control period to 5.8 (IQR: 2.2) items in the intervention period. The absolute number of daily purchased healthier snacks per 1000 customers during the placement + price intervention was 2.7 times higher (SE: 1.2, 95% CI: 2.0–3.6; 110) = 6.9, *p* < 0.001), as compared to the control period. No statistically significant difference in effect was found between the placement and the placement + price intervention with regard to the increase in the absolute number of daily purchased healthier snacks per 1000 customers between the intervention and control periods (ratio: 1.1, SE: 1.3, 95% CI: 0.7–1.9; t(29) = 0.2, *p* = 0.8). Furthermore, the median sales per 1000 customers per day of the less healthy snacks increased from 15.4 (IQR: 1.8) items in the control period to 18.1 (IQR: 1.4) items in the intervention period (including both placement and placement + price intervention). The absolute number of daily purchased less healthy snacks per 1000 customers was 1.3 times higher (SE: 1.1) in the intervention period (including both placement and placement + price), as compared to the control period (95% CI: 1.1–1.5; t(40) = 3.8, *p* < 0.001). The per-protocol analyses revealed similar results (data not shown).

## 3. Discussion (Study 1 and Study 2)

The first study indicate that completely substituting less healthy snacks with healthier alternatives at supermarket checkouts resulted in an overall decrease in the number of purchased checkout snacks, indicating that customers did not replace less healthy snack purchases with healthier alternatives. The second study indicate that placing additional healthier snacks at the checkouts, while keeping the less healthy snacks in place, did not lead to a lower purchase of less healthy snacks, that even slightly increased. However, the purchase of healthier snacks did not increase when they were additionally discounted. Thus, positioning healthier snacks at the checkout counter, without removing less healthy snacks, did not result in the replacement of less healthy snack purchases with healthier alternatives. To discourage the purchase of less healthy snacks at supermarket checkouts, a total substitution of less healthy with healthier snacks is clearly the most effective.

In these two studies, we attempted to create a healthier environment in supermarkets by introducing healthier snacks at the checkout counters. The modest intervention effects and the limited purchase of healthier checkout snacks in both of the studies are similar to those obtained in previous studies [41,50,51,52]. For example, an experimental study conducted in supermarkets in Denmark showed that replacing unhealthy snacks with healthier options (e.g., fruit and vegetables) at a single checkout counter resulted in a modest effect on the sales of carrots, but not on the sales of any of the other healthier snacks that were provided at the checkout [41]. Another real-life experiment conducted in the Netherlands demonstrated that placing healthier snacks at checkout displays in a railway-station kiosk, while repositioning the unhealthy snacks and displaying them elsewhere in the kiosk, increased healthier snack sales with 2 percent point (from 4% to 6% per day). This translates into a slight daily increase of approximately 10 healthier snacks per day [51]. Previous studies have reported results similar to those obtained in Study 2, in which purchases of unhealthy snacks were not replaced by purchases of healthier options [25,53,54]. It is important to note that, in Study 2, fewer healthier snacks were available relative to the large assortment of less healthy snacks at the checkouts. Moreover, the displays containing the healthier snacks were placed in front of the checkout counters, while the less healthy snacks were located at the conveyor belts. As a result, customers had limited exposure to the healthier snacks and were exposed to the less healthy snacks for a longer time. The exposure of the healthier snacks was maybe too low to cause a substitution effect. Taken together, the results of this and other studies suggest that placing healthier snacks at supermarket checkouts, while leaving the unhealthy snacks in place, is not sufficient to steer customers towards making healthier food choices at the checkout counter.

The results of this study provide further support for the idea that removing less healthy snacks from supermarket checkout counters is likely to decrease some unhealthy food purchases in supermarkets. As demonstrated in a previous study, food policies in supermarkets aimed at limiting unhealthy food products at checkout counters that were introduced voluntarily by six major supermarket chains in the United Kingdom, resulted in a reduction of purchases of common unhealthy checkout snacks. Moreover, this reduction was sustained over a year [23]. Despite the reduction in the overall sales of checkout snacks observed in both our Study 1 and this previous study, limiting the assortment of unhealthy checkout snacks is an efficient action to create a retail environment that supports healthier food choices, and in turn can benefit population health. It is nevertheless important to consider the financial consequences of such interventions for supermarkets. A reduction in the sales of checkout snacks might pose an economic risk that supermarkets are not willing to accept. Re-arranging the checkout environment to promote healthier food choices might require a different business model, and this should be considered when implementing such interventions in supermarkets. Encouraging supermarkets to find substitute checkout products (e.g., toothpaste or other non-food products) that would protect profit margins without having a potentially adverse impact on the health of consumers could be worth considering.

Our studies are subject to a number of strengths and limitations. A major strength was that we conducted real-life experiments. In doing so, we intervened at a key point of purchase for many people. In addition, we used sales data from supermarkets to measure food purchases, thus resulting in objective data that have been shown to provide a reasonably accurate measure of overall dietary quality [55,56]. It is, nevertheless, important to note that food purchases do not necessarily reflect food consumption, which should be taken into account when considering the implications of our results. Another strength of our studies is that the interventions were conducted in urban areas with a lower SEP in order to reach people from groups with a lower SEP. This is of utmost importance since overweight and obesity and diet-related chronic diseases are more prevalent amongst them compared to people from groups with a higher SEP [57,58]. Furthermore, to our knowledge, Study 1 was the first research to examine the impact of replacing the entire assortment of unhealthy snacks from all of the checkout counters in a supermarket with healthier alternatives. Study 2 is also one of the first to combine a placement strategy with an additional discount at the checkout in order to promote healthy food purchases. Our studies therefore add relevant information to the growing body of literature on healthy checkout counter (HCC) interventions in supermarkets. Despite these contributions, our study is subject to several limitations. With respect to the first study, the healthier snacks were developed and selected by the supermarket chain, and they did not all meet the Guidelines of the Dutch Nutrition Centre [47]. They were, however, healthier options than those that were regularly offered for sale. In addition, we did not assess compliance in Study 1 due to insufficient personnel and we were therefore not able to assess if the intervention was delivered as conceived and planned. This could have led to potential over- or underestimation of the presented results in Study 1. With respect to the second study, the healthier checkout snacks were also offered at a second location in the supermarket, and we were not able to isolate the sales of these checkout products from their sales in other locations. Although an overall increase in the sales of healthier snacks is a desirable outcome from a public-health perspective, we cannot estimate the exclusive impact of a checkout counter intervention, as we did not measure the location in the supermarket from which the products were taken. Another limitation of our two studies has to do with internal validity, due to the challenges of conducting experiments in supermarkets. Although they did examine food purchases in a real-life setting, our studies were not conducted in a controlled setting, and the results are limited to three supermarkets within a short intervention period. We were, therefore, unable to consider seasonal variations and secular trends in food purchases, nor could we control for such time-dependent factors. Future studies should include a larger sample of supermarkets and investigate the effects of such interventions over a period. Furthermore, because we did not have access to individual-level purchase data, we were not able to investigate the effects of the interventions at the household or individual level. For example, the results of our studies cannot be used to assess whether customers were triggered to select healthier options over unhealthy snacks or to purchase additional healthier snacks at the checkout counter. To generate further insight into this matter, future studies should examine changes in individual food purchases. Another limitation of both studies was that we were not able to collect information on the opening and closing hours of the different checkouts so we could not control for it in our analysis. Therefore, potential overestimation of the presented result cannot be excluded. However, this is one of the consequences of conducting experiments in a real-life setting and that future studies should account for. A final limitation of our research is that the results cannot be generalized as only three supermarkets were included in this research.

It is known from the literature that supermarket checkout counters are a place of impulse purchases and that these impulse purchases are usually unhealthy [36]. Our studies showed that placing and discounting additional healthier snacks at supermarket checkouts is not very effective in steering healthier over less healthy purchases. From a theoretical point of view, this suggests that impulsive food decisions are not only driven by the location where impulse purchases are made (e.g., at checkouts) but also by product characteristics that trigger impulsive decisions (healthier vs. less healthy). It could be that food decisions that are automatic and are steered by environmental cues (System 1 according to the dual process theory) are mainly triggered by energy-dense foods, as a result of our evolutionary preferences for foods high in sugar, salt and fat [59]. Our mind could just not be triggered by the characteristics of healthier products, and thus not resulting in an impulsive purchase response when facing these snacks at supermarket checkouts.. Furthermore, price is a key determinant of food choices, and has been shown to have precedence over other determinants of food choice, especially for people from groups with a low SEP. [19,23,24,60]. In our studies the price of healthier snacks may not have been optimized to compete with less healthy snacks: i.e., the healthier snacks may have been perceived as too expensive. Further research into the perceived value and optimal corresponding price for healthier snacks should lead to better strategies of making healthier snacks more competing with the less healthy snack assortment.

Creating healthier food environments is one of the goals of the National Prevention Agreement (NPA) to prevent obesity and was initiated by the Dutch Ministry of Health, Welfare and Sport presented in 2018. The NPA describes that supermarkets should tempt their customers to purchase more healthy products [61]. A recent report shows that Dutch supermarkets only limited adhere to the goals of the NPA [62]. From a societal point of view, our results are in line with these observations and indicate that retail efforts to promote healthier food choices at the checkouts in supermarkets are insufficient to reduce the purchase of less healthy products. As no evidence has revealed yet that removing less healthy products from one point of purchase (e.g., at supermarket checkouts) could result into compensatory effects (e.g., purchasing a candy bar) at other point of purchases, it could be assumed that removing less healthy snacks from supermarket checkouts would reduce total unhealthy food consumption. A direction that requires further investigation is the implementation of a policy action that actively enforces supermarkets to limit or ban less healthy products from impulsive points of purchase, like checkouts.

The introduction of healthier snacks at supermarket checkout counters is practically feasible, requires little effort to implement and could be one factor contributing to the promotion of healthier food choices in supermarkets. Nevertheless, a healthier checkout environment alone might be too limited to have any substantial impact on overall food purchases in supermarkets, especially given the fact that the majority of the food products available in supermarkets are unhealthy [28,63,64]. In addition to the checkout environment, other places within the supermarket (e.g., the ends of aisles, shelves at eye level and island bins) are designed to increase impulsive purchases and are predominantly used for unhealthy foods [3,28,36,65]. Future studies should therefore assess a healthy checkout counter as part of a multi-component intervention to promote healthier food purchases during the entire supermarket shopping experience. Previous reviews have indicated that supermarkets should combine effective health interventions that are implemented simultaneously in order to create an environment that supports healthy food choices [26,31]. Moreover, spillover effects of placing and discounting healthy products (e.g., at supermarket checkouts) on healthier purchases in other settings should additionally be investigated.

In recent years, brick-and-mortar food purchases have been shifted towards online purchases, while supermarkets without checkout counters grew their presence [66,67,68]. These trends are expected to even further grow in the near future. Both trends have a reduced impact on the availability of unhealthy impulse purchases at checkouts. Although, other important points of impulsive purchases (e.g., top shelves, ends-of-aisles) will remain or arise (e.g., via the online supermarket home page) and might even grow in importance for supermarket revenue strategies. Future research could attribute to the understanding of these developments and its impact on healthy and impulse food purchases.

## 4. Conclusions

The findings highlight that if supermarkets want to promote healthier snack purchases, healthier products can be positioned and discounted near the checkout counters. Yet, to discourage less healthy snack purchases at supermarket checkouts, a total substitution of less healthy with healthier alternatives (or just removing less healthy snacks) is clearly the most effective. Future studies should assess the feasibility of healthy checkout policies and finding substitute (non-food) products to protect both profit margins and consumer health. Moreover, a healthy checkout counter should be investigated as part of a multi-component intervention to promote healthier food purchases during the entire supermarket shopping experience.

## Figures and Tables

**Table 1:** Healthy Checkout Counter (HCC) intervention (period, type of healthier snacks and type of intervention (placement vs. placement + price) for each supermarket separately).

Period	Week	Type of Healthier Snacks at Checkout Counters	Type of Intervention at Supermarket 1	Type of Intervention at Supermarket 2
Control	1–2	X	X	X
Intervention	3–4	Cherry tomatoes and snack cucumbers	Placement	Placement + price
Intervention	5–6	Five kinds of unsalted nuts	Placement + price	Placement + price
Intervention	7–8	Snack tomatoes and snack bell peppers	Placement + price	Placement

**Table 2:** Effect of the Healthy Checkout Counter (HCC) intervention on the purchase of healthier snacks and less healthy snacks at checkout counters per 1000 customers per day (placement, placement + price intervention separately) between the intervention period and the control period.

	t-Value	Ratio (SE)	95%CI ^1^
Healthier snacks (placement)	3.2	2.1 (1.3) *	1.3–3.3
Healthier snacks (placement + price)	6.9	2.7 (1.2) *	2.0–3.6
Healthier snacks (placement + price vs. placement	0.2	1.1 (1.3)	0.7–1.9
Less healthy snacks (as usual at checkout counters)	3.8	1.3 (1.1) *	1.1–1.5

^1^ Independent-sample t-tests performed on log-transformed data; ratio is the exponent of the log-transformed outcome. * *p* < 0.05. SE = standard error. CI = confidence interval.
